# Digital Weight Loss Intervention for Parents of Children Being Treated for Obesity: A Prospective Cohort Feasibility Trial

**DOI:** 10.2196/11093

**Published:** 2018-12-20

**Authors:** Melissa C Kay, Jasmine Burroughs, Sandy Askew, Gary G Bennett, Sarah Armstrong, Dori M Steinberg

**Affiliations:** 1 Duke Global Digital Health Science Center Duke Center for Childhood Obesity Research Duke University Durham, NC United States; 2 Duke Global Digital Health Science Center Duke University Durham, NC United States; 3 Duke Global Digital Health Science Center Duke Department of Psychology and Neuroscience Duke University Durham, NC United States; 4 Duke Center for Childhood Obesity Research Duke Department of Pediatrics Duke University Durham, NC United States; 5 Duke Global Digital Health Science Center Duke School of Nursing Duke University Durham, NC United States

**Keywords:** obesity, parent, child, digital weight loss intervention, self-monitoring, weight, text

## Abstract

**Background:**

The prevalence of childhood obesity continues to increase, and clinic-based treatment options have failed to demonstrate effectiveness. One of the strongest predictors of child weight is parent weight. Parental treatment for weight loss may indirectly reduce obesity in the child. We have previously demonstrated the effectiveness among adults of a fully automated, evidence-based digital weight loss intervention (Track). However, it is unknown if it is feasible to deliver such a treatment directly to parents with obesity who bring their child with obesity to a weight management clinic for treatment.

**Objective:**

The objective of our study was to evaluate the feasibility of and engagement with a digital weight loss intervention among parents of children receiving treatment for obesity.

**Methods:**

We conducted a 6-month pre-post feasibility trial among parents or guardians and their children aged 4-16 years presenting for tertiary care obesity treatment. Along with the standard family-based treatment protocol, parents received a 6-month digital weight loss intervention, which included weekly monitoring of personalized behavior change goals via mobile technologies. We examined levels of engagement by tracking completed weeks of self-monitoring and feasibility by assessing change in weight.

**Results:**

Participants (N=48) were on average 39 years old, mostly female (35/42, 82% ), non-Hispanic Black individuals (21/41, 51%) with obesity (36/48, 75%). Over a quarter had a yearly household income of <US $25,000, and about a third had the equivalent of a high school education. Children were on average 10 years old and had a body mass index of 29.8 kg/m^2^. The median percentage of weeks participants tracked their behaviors was 77% (18.5/24 total weeks; interquartile range [IQR] 6.3 to 100). The median number of attempts via phone or text message (short message service) required to complete one tracking week was 3.3 (IQR 2.6 to 4.9). Nearly half (23/48, 48%) had high levels of engagement, completing 80% (19/24) or more weeks of tracking. Of the 26 participants with weight measurements reported at 6 months, of which 81% (21/26) were self-reported, there was a median 2.44 kg (IQR −6.5 to 1.0) decrease in weight.

**Conclusions:**

It is feasible to deliver an evidence-based digital weight loss intervention to parents or guardians whose children are enrolled in a weight management program. Given the feasibility of this approach, future studies should investigate the effectiveness of digital weight loss interventions for parents on child weight and health outcomes.

## Introduction

The prevalence of obesity among children has increased since 1999, and rates among non-Hispanic black and Hispanic children are consistently higher compared with non-Hispanic White children [1-3]. Children with obesity are at increased risk of developing chronic conditions during childhood and during adulthood if obesity persists [4-8]. Children of racial or ethnic minority are disproportionately affected both in terms of obesity and chronic disease.

Recent recommendations from the US Preventive Services Task Force suggest children aged 6 and older with obesity be referred to intensive lifestyle-based weight loss programs [9]. These require 26 or more hours of provider contact with greater effectiveness demonstrated with more contact hours and the incorporation of behavior change techniques such as goal setting and self-monitoring [10]. Although children and parents report positive experiences in behavioral weight loss programs, logistical issues such as clinic hours and location and required time commitment lead to discontinuation of care [11,12]. These high levels of attrition have resulted in poor efficacy [13]. Thus, innovative approaches to pediatric weight management are necessary.

Obesity is highly comorbid in families [14,15]. Although family-based interventions are effective in reducing child body mass index (BMI) [16], they can be time intensive and costly [17]. Yet, parent-only interventions have been effective in the treatment of childhood overweight and obesity [18-20]. Indeed, parent weight change is a strong predictor of child weight change [21,22], in that a 1-unit reduction in parent BMI is associated with a 0.26 reduction in child BMI after participation in a behavioral weight loss program [23]. Because the child weight status is associated with the parent weight status [24-26], parental treatment for weight loss may indirectly reduce obesity in the child by impacting the family’s shared environment and through parental role modeling of healthy behaviors. Although pediatric obesity management programs include discussion on changing family behaviors, most programs do not directly and independently treat the parent’s obesity. Innovative strategies are needed to consider how best to treat parental obesity while treating children with obesity. Digital health interventions may be well suited to achieve this goal [27].

Digital health approaches capitalize on the ubiquitous utilization of mobile technologies [28], and they have great potential to be scalable and integrated into the existing clinical infrastructure (eg, electronic health records). Digital approaches overcome barriers to parental involvement in weight management programs, such as the time required for attendance and childcare, because they can be asynchronous with care (ie, delivered without requiring real-time interaction). Prior work demonstrates that using mobile technologies to administer weight loss treatment can be successful in the clinic setting [29,30]. We recently demonstrated the effectiveness of “Track,” a fully automated, evidence-based digital weight loss program, among adults in a clinic setting [31,32]. In a similar intervention, participants who engaged more, as measured by self-monitoring of behaviors associated with weight loss, lost more weight [33]. Others have demonstrated the importance of user engagement leading to optimal behavior change [34-38]. Measuring engagement is an important measure of fidelity, ensuring that treatments are delivered in the dose intended [39]. Thus, the primary aim of this feasibility study was to measure user engagement, as measured by self-monitoring, after delivering Track to parents or guardians of children with obesity who are presenting for weight management. Assessing feasibility and engagement will aid in determining how best to design future intervention studies.

## Methods

### Study Design

We delivered a 6-month pre-post feasibility trial called Families on Track to parents or guardians of children seeking treatment for weight management. We recruited participants from the Duke Healthy Lifestyles clinic. Healthy Lifestyles is a referral-based pediatric weight management program located in Durham, NC, which serves a population that is racially and ethnically diverse; 57% are female, 61% are black individuals, 29% are Hispanic individuals, and 70% of patients have public health insurance. The Healthy Lifestyles clinical protocol, patient demographics, and outcomes have been previously described [11,13], and the program represents the current standard of care for obesity treatment. All participants received the Healthy Lifestyles intended clinical treatment protocol. The Duke Medical Center Institutional Review Board approved all procedures.

### Participants

Participants included parents or guardians of children aged 4-16 years with an age- and gender-specific BMI of ≥95th percentile presenting for obesity treatment to the Duke Healthy Lifestyles clinic. Eligibility criteria included parents or guardians aged 18-60 years with BMI between 25 and 50 kg/m^2^. We required that participants have English fluency, own a mobile phone and be willing to send and receive multiple short message service (SMS) text messages per day, and reside in the same household as the patient attending Healthy Lifestyles. We excluded participants who were pregnant or lactating; had prior or planned bariatric surgery; were participating in other obesity trials; had a history of heart attack, stroke, bipolar disorder, schizophrenia or recent cancer diagnosis; or had plans to relocate within 1 year. We recruited a convenience sample of 50 participants; 2 were excluded (1 did not meet BMI criteria and 1 declined). A total of 48 participants were consented and enrolled by a trained research assistant, who then collected baseline data.

### Intervention

The Families on Track intervention included the Healthy Lifestyles program plus a 6-month modified version of Track, a digital weight loss intervention for adults that was conducted in the primary care setting. The Healthy Lifestyles program has been described in detail elsewhere [13]. Briefly, the Healthy Lifestyles program uses best-practice pediatric weight management strategies, which involves monthly visits for patients and their families with medical, dietary, and exercise specialists all certified in Motivational Interviewing. Patients set dietary and activity behavioral goals aimed to improve the severity of overweight or obesity and obesity-related comorbidities. The Track intervention, summarized elsewhere [31], was modified to contain 4 components: tailored behavioral goals (eg, no sugary drinks, watch less than 2 hours of television per day, and walk 10,000 steps per day); self-monitoring of these goals via interactive voice response (IVR) phone calls or SMS text messages; skills training videos; and an analog bathroom scale and a pedometer to self-monitor daily weights and steps.

### Behavior Change Goals

The intervention utilized the Interactive Obesity Treatment Approach (iOTA), which results in weight loss through the modification of everyday obesogenic behaviors [29,30,40,41]. At baseline, each intervention participant completed a short, self-administered survey to assess the level of engagement in various dietary, physical activity, and other weight control behaviors. A computer algorithm used participant responses to assign personalized behavioral goals from a vast library of goals known to create an energy deficit (eg, *no sugary drinks, no fast food consumption*) based on each participant’s need to change each behavior, readiness and self-efficacy, and the potential caloric deficit promoted by the specific behavior change. The algorithm rank orders the goals, and participants are asked to self-monitor their adherence to the top 3 goals for the first 8 weeks of the study. Goals changed every 8 weeks throughout the 24-week intervention period to maintain motivation and facilitate goal mastery. Participants also received a universal 4th goal. We assigned a “*no red zone foods* ” goal for the first 8 weeks. To determine the “red zone foods,” we asked participants to select the foods they consume regularly (at least 3 days per week) from a list of commonly eaten, high-calorie foods and beverages (eg, sodas, sweet tea, desserts, potato chips, pizza, and hamburgers). The other universal goals were to “*practice portion control*” and “*walk 7-10,000 steps per day.*” We provided all intervention participants with Web links to a study-specific YouTube channel that included descriptive and skills training videos specific to each Track goal. We reminded participants to refer to the videos for additional skills training and behavior change tips, specifically when goals changed every 8 weeks.

### User Engagement

**Figure 1:**
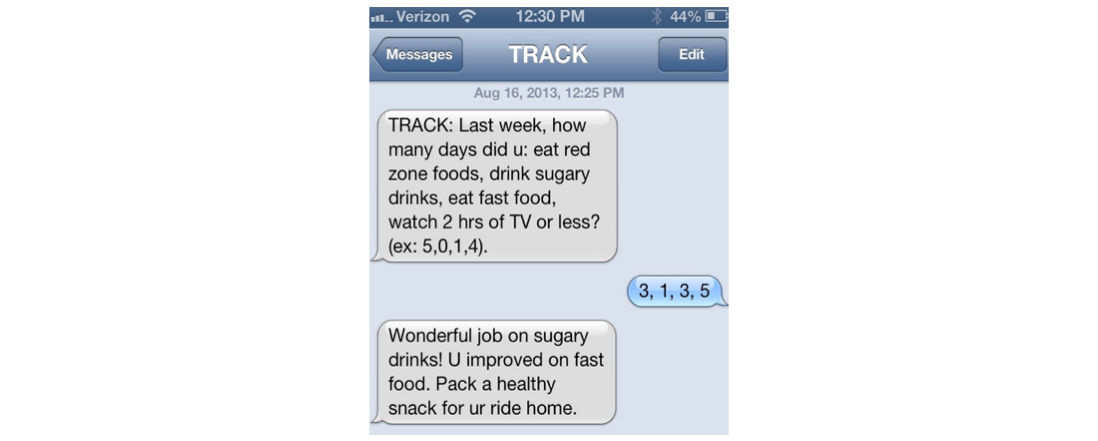
Example of a self-monitoring SMS text message sent weekly to participants in Families on Track (Interactive Obesity Treatment Approach [iOTA], Duke Global Digital Health Science Center).

We measured user engagement with the intervention both quantitatively and qualitatively. Using quantitative measures, we tracked the frequency of weekly self-monitoring across the 24-week intervention. Participants were expected to self-monitor daily via paper log and weekly using the IVR system or through SMS text message. Each week, participants received an automated prompt from the Families on Track intervention system to track adherence to behaviors goals. These prompts were delivered either via IVR or SMS text message. The IVR system called intervention participants weekly to request self-monitoring data and provided automated tailored feedback on the 4 goals. Participants who did not respond to IVR attempts were sent a SMS text message prompting them to communicate their weekly tracking data via SMS text messages (Figure 1). Participants who provided self-monitoring data via SMS text messaging also received tailored feedback. We have a robust retry protocol that attempts to reach participants if the first IVR call or SMS text message goes unanswered. Tracking was considered complete if the participant completed the entire weekly IVR call or responded to the weekly SMS text message. User engagement with the intervention was assessed by totaling the number of weeks each participant responded to prompts to track behavior across the 24 weeks. In addition, we created a dichotomous outcome variable to compare those who were high versus low engagers using an established cutoff of 80% or more engagement in weekly self-monitoring [33,42]. We also tracked the mean number of prompts required to elicit a response for each participant as an additional measure of user engagement. For a qualitative measure of user engagement, participants were asked to complete satisfaction surveys upon study completion to assess the acceptability of the message frequency, timing, content, and perceived usefulness. Prior to their 6-month follow-up, participants were prompted to complete the satisfaction survey. Attempts were made via phone, email, and SMS text messages to complete the survey even if a follow-up appointment could not be scheduled.

### Weight

A trained nurse in the Healthy Lifestyles clinic collected parent and guardian height and weight at baseline and at 6 months; we measured height using a stadiometer (Model: Healthometer Professional CE No 92977) and weight using a digital scale (Model: Seca CE No 96990). A high percentage of participants did not return to the Healthy Lifestyles clinic for follow-up appointments despite email, phone, and SMS text message reminders. Therefore, we experienced difficulties in scheduling the 6-month visits. Thus, we also collected weights via self-report. Self-reported weights were sent to the study staff via email, SMS text message, or phone from participants who were unable to complete their in-clinic study visit. To verify self-reported weights, participants were asked to SMS text message or email the study staff a photo of their feet on their study-issued or personal scale with a visible weight reading.

### Other Measurements

Sociodemographic variables were measured using standard questionnaires completed by the parent or guardian at the baseline clinic visit.

### Analysis

We used descriptive statistics to characterize participants and examine tracking completion rate and weight change over the 6-month period. Characteristics were summarized using frequencies and proportions for categorical variables and mean (SD) for continuous variables. We used medians and interquartile ranges (IQR) to summarize intervention engagement and weight change owing to its highly skewed distribution. We conducted bivariate analyses to examine potential predictors of intervention engagement using Wilcoxon-Mann-Whitney and Kruskal Wallis tests for continuous data and chi-square and Fisher’s exact tests for categorical data. We used Poisson regression with a robust variance to examine sociodemographic differences among those with higher levels of tracking engagement (80% or more weeks of tracking) and estimate risk ratios (RRs) and 95% CIs. To assess intervention feasibility, we assessed differences in weight change among high and low engagers using the Wilcoxon-Mann-Whitney test. We conducted all analyses using Stata 14 for Mac (StataCorp, College Station, TX) with an alpha value of <.05 to assess statistical significance.

## Results

### Baseline Characteristics

At baseline (N=48), participants were on average 39.4 years old (SD 7.3) with a mean BMI of 36.5 kg/m^2^ (Table 1). Half (21/41, 51%) were non-Hispanic black individuals. Most were female (35/42, 83%) and the mother of the child (34/41, 83%) and many were employed (33/41, 81%). Over a quarter, 26% (10/38) had an income of <US $25,000, and the highest level of education for over a third of participants (13/37, 35%) was a high school equivalent.

### User Engagement

**Figure 2:**
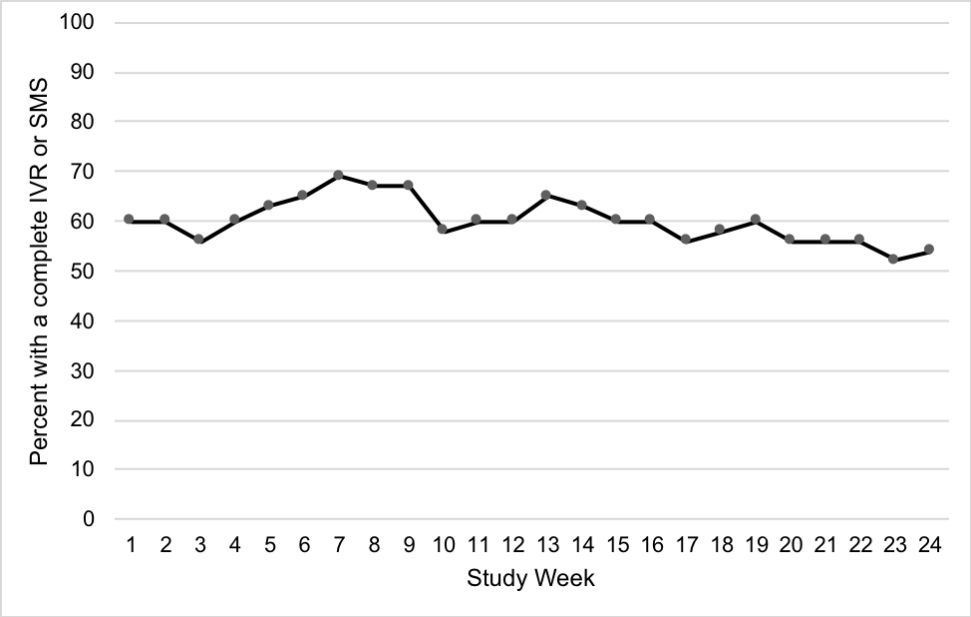
Proportion of participants with a complete tracking week as measured by a completed IVR call or SMS text message, by study week (N=48). IVR: interactive voice response; SMS: short message service.

At least half of the participants engaged in tracking their behaviors in each study week, as measured by a complete IVR call or SMS text message (Figure 2). The median engagement rate was 77% (IQR 6.3 to 100) across all study weeks. A fifth of participants (10/48, 21%) did not track any of their behaviors, and 27% (13/48) completed all tracking weeks. Nearly half (23/48, 48%) of the participants were considered high engagers (based on a median split), tracking their behaviors for at least 80% (19/24) of study weeks. The median number of prompts required to get participants to complete 1 tracking week (either through IVR or SMS text messages) was 3.3 (IQR 2.6 to 4.9). Most of the tracking was completed via SMS text messages (87%) than with IVR. The average duration in minutes for those who did complete IVR calls was 0.5 (SD 0.9).

Among the included participants, 40% (19/48) completed the post intervention satisfaction survey. Those with complete surveys completed more weeks of tracking, 21.3 (SD 3.8) versus 9.9 (SD 10.2) with *P*<.001, and were more likely to be high engagers (*P*=.005). All respondents (19/19, 100%) felt it was easy to understand their 4 Track goals, among whom 89% (16/18) felt confident they could follow them and 89% (17/19) felt they were what they needed to work on to lose weight. Most (16/19, 84%) liked being able to choose each week whether they responded to tracking requests via SMS text messages or IVR. A few felt the weekly automated calls (5/19, 26%) and texts (2/19, 11%) were difficult to understand, but most (16/19, 84%) felt the feedback received on them was helpful. About a quarter of the respondents (5/19, 26%) found getting started in Track was hard. Of those receiving SMS text messages (10/19, 53%), most reported the number of texts was just enough.

**Table 1:** Baseline characteristics of parents or guardians participating in a digital weight loss intervention.

Characteristics at enrollment			Value
**Parent or guardian characteristics**			
	**Race or ethnicity (N=41), n (%)**		
		Non-Hispanic black	21 (51)
		Hispanic	5 (12)
		Non-Hispanic white	11 (27)
		Other	2 (5)
		Declined	2 (5)
	Age (N=42), mean (SD)		39.4 (7.9)
	**Gender (N=42), n (%)**		
		Female	35 (83)
	**Relation to the child (N=41), n (%)**		
		Mother	34 (83)
		Father	6 (15)
		Grandmother	1 (2)
	Employed (N=41), n (%)		33 (81)
	**Income level (N=38), n (%)**		
		<US $25,000	10 (26)
		US $25,000-34,999	13 (34)
		≥ US $35,000	15 (40)
	**Education (N=37), n (%)**		
		High School equivalent	13 (35)
		Tech or community college	9 (24)
		College degree or more	15 (41)
	Married (N=41), n (%)		21 (51)
	Household size (N=42), mean (SD)		4.2 (1.3)
	BMI^a^ (N=48), mean (SD)		36.5 (8.0)
**Child characteristics**			
	**Race or ethnicity (N=48), n (%)**		
		Non-Hispanic black	25 (52)
		Hispanic	6 (13)
		Non-Hispanic white	9 (19)
		Pacific Islander	1 (2)
		Other	5 (10)
		Declined	2 (4)
	Age (N=48), mean (SD)		10.0 (3.4)
	**Gender (N=48), n (%)**		
		Female	27 (56)
	BMI (N=48), mean (SD)		29.8 (7.9)

^a^BMI: body mass index.

### Predictors of User Engagement

**Figure 3:**
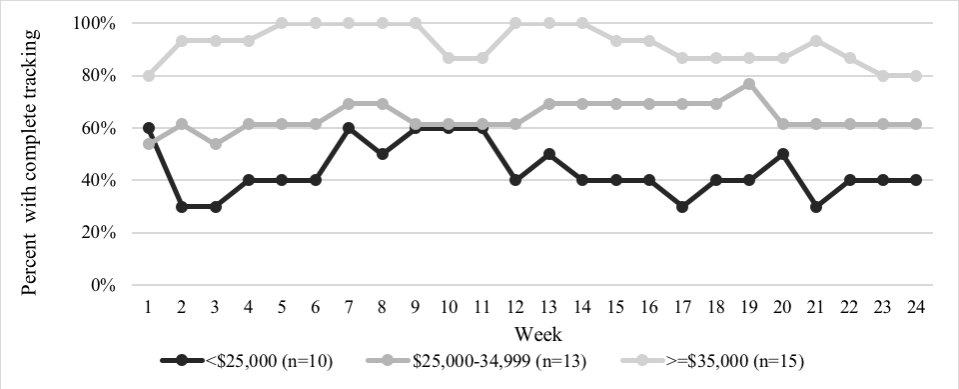
Proportion of participants with a complete tracking week as measured by a completed IVR call or SMS text messages, by study week and income level (N=38).

**Figure 4:**
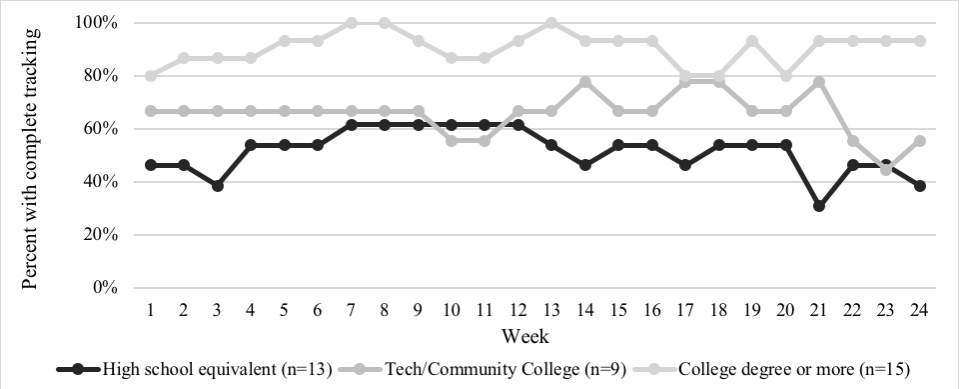
Proportion of participants with a complete tracking week as measured by a completed IVR call or SMS text message by study week and education level (N=37).

The percent of tracking weeks completed was positively associated with education and income (*P*=.01 and *P*<.001, respectively; Figures 3 and 4). Income and parent race or ethnicity were associated with level of engagement. Participants with incomes >US $35,000 per year were 4 times as likely to be high engagers (ie, completed >80% of tracking weeks) compared with those with incomes <US $25,000 (RR 4.0; 95% CI 1.1-14.4; *P*=.03); this relationship was attenuated after controlling for parent race or ethnicity, though remaining significant (RR 3.5; 95% CI 1.1-11.4; *P*=.04). Non-Hispanic White individuals were twice as likely to be high engagers (RR 2.1; 95% CI 1.2-4.0; *P*=.02) compared with non-Hispanic black individuals, though this relationship was not significant when controlling for income (RR 1.5; 95% CI 0.8-2.6; *P*=.19). As child age increased, participants were less likely to be high engagers (RR 0.9; 95% CI 0.8-1.0; *P*=.04).

### Weight Change

**Figure 5:**
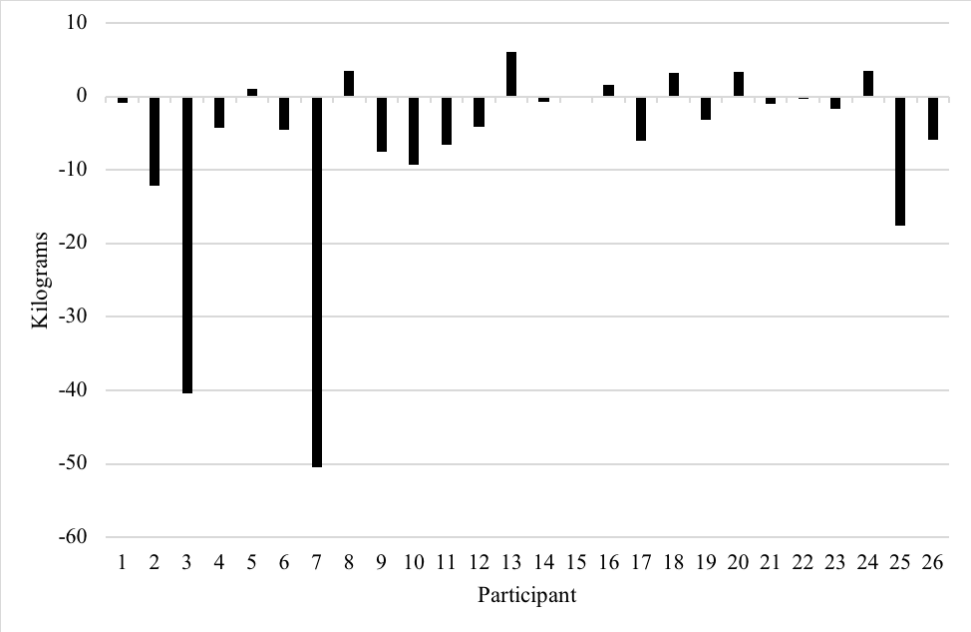
Change in weight among participants with complete pre and post weight data participating in a digital behavioral weight loss intervention (N=26).

At the 6-month intervention completion point, weight was recorded from 54% (26/48) of the participants. Of those, 81% (21/26) were self-reported. There were no significant sociodemographic differences among those with a self-reported weight versus those who were missing weight measurements at 6 months. Those who reported weight at 6 months tracked significantly more weeks than those who did not report weight, 17.3 (SD 8.7) versus 11.0 (SD 10.4), *P*=.03; however, they were not more likely to be high engagers (*P*=.14). Of the 26 participants with complete pre and post intervention weight data, there was significant median weight loss of 2.44 kg (IQR −6.5 to 1.0; *P*=.01; Figure 5). Many (18/26, 69%) had a net weight loss, whereas few (7/26, 27%) had a net weight gain. There was no difference in weight change among high and low engagers. We conducted a sensitivity analysis to exclude those with self-reported weight loss of >40 kg (N=2); the results remained significant with a median weight loss of 1.3 kg (IQR −6.0 to 1.3; *P*=.04).

## Discussion

### Principal Findings

These findings suggest that we can feasibly recruit and engage parents or guardians who are attending a pediatric weight management program for their child with obesity in a digital weight loss intervention. Almost half of the participants (23/48, 48%) were high engagers, tracking behaviors for 80% (19/24) or more study weeks. What is most notable about this study is the parent focus. We aimed to recruit a sample of parents who were interested in obesity treatment for their children, but what we found is that some parents did not engage in self-monitoring of behaviors that result in weight loss for themselves, despite presenting for treatment for their children. We were able to recruit and engage parents, but we had difficulties retaining them and asking them to complete study assessments for evaluation. However, our study did demonstrate favorable behavioral outcomes. Most of the participants who reported weight upon study completion experienced weight loss and found the intervention easy to participate in with accurate goals and helpful feedback. Although this study was not designed to establish efficacy, it is promising that among this group of participants with relatively high engagement, there was significant weight loss.

The results from the Families on Track study are similar to what we found in the Shape Plan trial, which aimed to test the feasibility of delivering daily SMS text messages tracking behavioral goals [41]. In that study, we found that 85% tracked at least 2 days per week and the average weight loss was around 2 kg after 6 months [29]. Finding similar feasibility and weight loss findings suggest that a standalone approach to weight loss that focuses primarily on tracking behavioral goals through mobile technologies can be effective for parents of children presenting for obesity treatment or other adult populations.

Our findings are comparable to reported engagement outcomes from other similar behavioral interventions using SMS text messaging or other digital health modalities. Among breast cancer survivors who were overweight and participated in a 10-week multifaceted mobile health study, engagement with SMS text messaging was 86% [43]. In a year-long childhood obesity reduction intervention targeting parents and their children, 66% of parents were considered high completers for SMS text message response rates [44]. A unique aspect to the Track system, which likely contributed to high engagement, is its ability to provide fully automated, tailored feedback based on participant behaviors [45]. Many intervention studies have relied on one-way SMS text messaging delivering less personalized, more static content. Engagement and effectiveness can be increased by adding other components, such as the provision of human support, but requires greater cost and intensity [46]. That greater cost and intensity may not be feasible to deliver to parents with children presenting for obesity treatment.

Studies show that mobile phones can be an effective tool in weight loss interventions, given the increased ease in self-monitoring behaviors compared with using typical paper logs [47]. Participant engagement in Families on Track was largely completed via SMS text messages than with IVR, which was contrary to similar studies in which IVR was the preferred modality [48]. Parents might find it easier to engage in SMS text messages given they can respond at a time that suits them and are provided visual feedback, which they can retain and refer to.

Involving parents in weight-related behavior change interventions has demonstrated effectiveness in reducing child overweight or obesity [18-20]. However, the best way to support parents of children with obesity is not well known. Few pediatric weight management clinics or organizations have the resources to provide a parent-only approach in addition to childhood obesity treatment. Most pediatric weight management clinics are not well equipped to care for adult health. Additionally, parents are not uniformly engaged in their own weight management when they bring their child to weight management programs, making it difficult to determine the most generalizable way to engage parents. Future studies are needed to determine the best way to engage parents of children with obesity in a way that meets various levels of motivation without high burden. Targeting parents based on characteristics associated with higher levels of engagement and feasibility may be the best approach. We found that parents of older children were less likely to be highly engaged, meaning effective treatment strategies can vary by the age of the child. Family-based interventions may be more effective for parents of older adolescents because these children are more autonomous and make many of their own decisions regarding food choice. They are also able to help when it comes to cooking and meal planning.

Our study was not immune to the disparities or inequalities in engagement seen in other digital health interventions [33,48]. Although overall participants were of lower socioeconomic status, parents or guardians with higher levels of education and income demonstrated higher engagement. This speaks to the importance of designing digital weight loss interventions that are adapted to the needs and habits of various social groups, particularly those most vulnerable and at risk for obesity. Studies show that it is possible to reach and engage more socioeconomically disadvantaged populations with a lower intensity digital health intervention [49], but more work needs to be done to ensure a broader reach and consistent engagement concurrent with positive behavioral outcomes.

### Limitations

Limitations to our feasibility study include a small sample size and lack of a control group. We felt it would be difficult to withhold treatment from parents who are already presenting for their child’s treatment. An additional limitation is that parents or guardians that attend tertiary care clinics for their children may have different motivations, especially given they are presenting for their children and not themselves. Future studies are needed to assess true generalizability among parents within the general population and also within primary care clinics. Although the results of this study show promise having achieved high engagement, more research is needed to assess behavioral changes as a result of engagement in this population. A large limitation is that our weight change and qualitative user engagement data are not complete given the lack of returning clinic visits. We also collected postintervention weight primarily through self-report. As mentioned earlier, we provided this option because it was difficult to have participants return for assessment visits. This is likely because of the way the Healthy Lifestyle program is structured—children do not present for treatment often after the initial treatment is provided in the first month. It may be that parents were not interested in attending if their children were not attending for their own treatment. As a result, it is possible our results are biased toward a larger effect. However, previous evidence does suggest that self-reported weights provide a reasonably accurate measurement among adults [50].

### Conclusion

In this feasibility study, we demonstrate that it is possible to engage parents or guardians of children with obesity in a digital weight loss intervention aimed at reducing parent weight. The digital intervention engaged a population of parents who are hard to reach through in-person visits and shows promise for reaching and engaging parents in future family-based obesity treatment interventions, an important aspect of intervention fidelity.

## Abbreviations

BMI: body mass index
iOTA: Interactive Obesity Treatment Approach
IQR: interquartile range
IVR: interactive voice response
RR: risk ratios
SMS: short message service

## References

[1] Ogden C, Carroll M, Fryar C, Flegal K (2015). Prevalence of Obesity Among Adults and Youth: United States, 2011-2014. NCHS Data Brief.

[2] Skinner AC, Perrin EM, Skelton JA (2016). Prevalence of obesity and severe obesity in US children, 1999-2014. Obesity (Silver Spring).

[3] Skinner A, Ravanbakht S, Skelton J, Perrin E, Armstrong S (2018). Prevalence of Obesity and Severe Obesity in US Children, 1999-2016. Pediatrics.

[4] Skinner AC, Perrin EM, Moss LA, Skelton JA (2015). Cardiometabolic Risks and Severity of Obesity in Children and Young Adults. N Engl J Med.

[5] Freedman D, Mei Z, Srinivasan S, Berenson G, Dietz W (2007). Cardiovascular risk factors and excess adiposity among overweight children and adolescents: the Bogalusa Heart Study. J Pediatr.

[6] Lloyd LJ, Langley-Evans SC, McMullen S (2012). Childhood obesity and risk of the adult metabolic syndrome: a systematic review. Int J Obes (Lond).

[7] Mohanan S, Tapp H, McWilliams A, Dulin M (2014). Obesity and asthma: pathophysiology and implications for diagnosis and management in primary care. Exp Biol Med (Maywood).

[8] Morrison K, Shin S, Tarnopolsky M, Taylor V (2015). Association of depression & health related quality of life with body composition in children and youth with obesity. J Affect Disord.

[9] Grossman David C, Bibbins-Domingo Kirsten, Curry Susan J, Barry Michael J, Davidson Karina W, Doubeni Chyke A, Epling John W, Kemper Alex R, Krist Alex H, Kurth Ann E, Landefeld C Seth, Mangione Carol M, Phipps Maureen G, Silverstein Michael, Simon Melissa A, Tseng Chien-Wen, US Preventive Services Task Force (2017). Screening for Obesity in Children and Adolescents: US Preventive Services Task Force Recommendation Statement. JAMA.

[10] O'Connor EA, Evans CV, Burda BU, Walsh ES, Eder M, Lozano Paula (2017). Screening for Obesity and Intervention for Weight Management in Children and Adolescents: Evidence Report and Systematic Review for the US Preventive Services Task Force. JAMA.

[11] Dolinsky D, Armstrong S, Østbye Truls (2012). Predictors of attrition from a clinical pediatric obesity treatment program. Clin Pediatr (Phila).

[12] Skelton JA, Martin S, Irby MB (2016). Satisfaction and attrition in paediatric weight management. Clin Obes.

[13] Dolinsky D, Armstrong S, Walter E, Kemper A (2012). The effectiveness of a primary care-based pediatric obesity program. Clin Pediatr (Phila).

[14] Lake JK, Power C, Cole TJ (1997). Child to adult body mass index in the 1958 British birth cohort: associations with parental obesity. Arch Dis Child.

[15] Garn SM, Sullivan TV, Hawthorne VM (1989). Fatness and obesity of the parents of obese individuals. Am J Clin Nutr.

[16] Berge JM, Everts JC (2011). Family-Based Interventions Targeting Childhood Obesity: A Meta-Analysis. Child Obes.

[17] Janicke DM, Steele RG, Gayes LA, Lim CS, Clifford LM, Schneider EM, Carmody JK, Westen S (2014). Systematic review and meta-analysis of comprehensive behavioral family lifestyle interventions addressing pediatric obesity. J Pediatr Psychol.

[18] Ewald H, Kirby J, Rees K, Robertson W (2014). Parent-only interventions in the treatment of childhood obesity: a systematic review of randomized controlled trials. J Public Health (Oxf).

[19] Yavuz HM, van Ijzendoorn MH, Mesman J, van der Veek S (2014). Interventions aimed at reducing obesity in early childhood: a meta-analysis of programs that involve parents. J Child Psychol Psychiatry.

[20] Niemeier B, Hektner J, Enger K (2012). Parent participation in weight-related health interventions for children and adolescents: a systematic review and meta-analysis. Prev Med.

[21] Wrotniak B, Epstein L, Paluch R, Roemmich J (2004). Parent weight change as a predictor of child weight change in family-based behavioral obesity treatment. Arch Pediatr Adolesc Med.

[22] Andriani H, Liao C, Kuo H (2015). Parental weight changes as key predictors of child weight changes. BMC Public Health.

[23] Boutelle KN, Cafri G, Crow SJ (2012). Parent predictors of child weight change in family based behavioral obesity treatment. Obesity (Silver Spring).

[24] Davis M, McGonagle K, Schoeni R, Stafford F (2008). Grandparental and parental obesity influences on childhood overweight: implications for primary care practice. J Am Board Fam Med.

[25] Liu Y, Chen H, Liang L, Wang Y (2013). Parent-child resemblance in weight status and its correlates in the United States. PLoS One.

[26] Bushnik T, Garriguet D, Colley R (2017). Parent-Child association in body weight status. Health Rep.

[27] Hammersley ML, Jones RA, Okely AD (2016). Parent-Focused Childhood and Adolescent Overweight and Obesity eHealth Interventions: A Systematic Review and Meta-Analysis. J Med Internet Res.

[28] (2015). Pew Research Center.

[29] Bennett GG, Foley Perry, Levine Erica, Whiteley Jessica, Askew Sandy, Steinberg Dori M, Batch Bryan, Greaney Mary L, Miranda Heather, Wroth Thomas H, Holder Marni Gwyther, Emmons Karen M, Puleo Elaine (2013). Behavioral treatment for weight gain prevention among black women in primary care practice: a randomized clinical trial. JAMA Intern Med.

[30] Bennett GG, Warner ET, Glasgow RE, Askew S, Goldman J, Ritzwoller DP, Emmons KM, Rosner BA, Colditz GA, Be FBWSI (2012). Obesity treatment for socioeconomically disadvantaged patients in primary care practice. Arch Intern Med.

[31] Foley P, Steinberg D, Levine E, Askew S, Batch BC, Puleo EM, Svetkey LP, Bosworth HB, DeVries A, Miranda H, Bennett GG (2016). Track: A randomized controlled trial of a digital health obesity treatment intervention for medically vulnerable primary care patients. Contemp Clin Trials.

[32] Bennett G, Steinberg D, Askew S, Levine E, Foley P, Batch B, Svetkey L, Bosworth H, Puleo E, Brewer A, DeVries A, Miranda H (2018). Effectiveness of an App and Provider Counseling for Obesity Treatment in Primary Care. Am J Prev Med.

[33] Steinberg DM, Levine EL, Lane I, Askew S, Foley PB, Puleo E, Bennett GG (2014). Adherence to self-monitoring via interactive voice response technology in an eHealth intervention targeting weight gain prevention among Black women: randomized controlled trial. J Med Internet Res.

[34] Strecher VJ, McClure J, Alexander G, Chakraborty B, Nair V, Konkel J, Greene S, Couper M, Carlier C, Wiese C, Little R, Pomerleau C, Pomerleau O (2008). The role of engagement in a tailored web-based smoking cessation program: randomized controlled trial. J Med Internet Res.

[35] Funk KL, Stevens VJ, Appel LJ, Bauck A, Brantley PJ, Champagne CM, Coughlin J, Dalcin AT, Harvey-Berino J, Hollis JF, Jerome GJ, Kennedy BM, Lien LF, Myers VH, Samuel-Hodge C, Svetkey LP, Vollmer WM (2010). Associations of internet website use with weight change in a long-term weight loss maintenance program. J Med Internet Res.

[36] Couper MP, Alexander GL, Zhang N, Little RJA, Maddy N, Nowak MA, McClure JB, Calvi JJ, Rolnick SJ, Stopponi MA, Cole JC (2010). Engagement and retention: measuring breadth and depth of participant use of an online intervention. J Med Internet Res.

[37] Donkin L, Christensen H, Naismith SL, Neal B, Hickie IB, Glozier N (2011). A systematic review of the impact of adherence on the effectiveness of e-therapies. J Med Internet Res.

[38] Sharpe EE, Karasouli E, Meyer C (2017). Examining Factors of Engagement With Digital Interventions for Weight Management: Rapid Review. JMIR Res Protoc.

[39] Bellg A, Borrelli B, Resnick B, Hecht J, Minicucci D, Ory M, Ogedegbe G, Orwig D, Ernst D, Czajkowski STFWOTNBCC, Treatment Fidelity Workgroup of the NIH Behavior Change Consortium (2004). Enhancing treatment fidelity in health behavior change studies: best practices and recommendations from the NIH Behavior Change Consortium. Health Psychol.

[40] Steinberg DM, Bennett GG, Askew S, Tate DF (2015). Weighing every day matters: daily weighing improves weight loss and adoption of weight control behaviors. J Acad Nutr Diet.

[41] Foley P, Levine E, Askew S, Puleo E, Whiteley J, Batch B, Heil D, Dix D, Lett V, Lanpher M, Miller J, Emmons K, Bennett G (2012). Weight gain prevention among black women in the rural community health center setting: the Shape Program. BMC Public Health.

[42] Vincze G, Barner JC, Lopez D (2004). Factors associated with adherence to self-monitoring of blood glucose among persons with diabetes. Diabetes Educ.

[43] Quintiliani LM, Mann DM, Puputti M, Quinn E, Bowen DJ (2016). Pilot and Feasibility Test of a Mobile Health-Supported Behavioral Counseling Intervention for Weight Management Among Breast Cancer Survivors. JMIR Cancer.

[44] Price S, Ferisin S, Sharifi M, Steinberg D, Bennett G, Wolin KY, Horan C, Koziol R, Marshall R, Taveras EM (2015). Development and Implementation of an Interactive Text Messaging Campaign to Support Behavior Change in a Childhood Obesity Randomized Controlled Trial. J Health Commun.

[45] Lyzwinski LN, Caffery LJ, Bambling M, Edirippulige S (2017). Consumer perspectives on mHealth for weight loss: a review of qualitative studies. J Telemed Telecare.

[46] Baumeister H, Reichler L, Munzinger M, Lin J (2014). The impact of guidance on Internet-based mental health interventions - A systematic review. Internet Interventions.

[47] Liu F, Kong X, Cao J, Chen S, Li C, Huang J, Gu D, Kelly TN (2015). Mobile phone intervention and weight loss among overweight and obese adults: a meta-analysis of randomized controlled trials. Am J Epidemiol.

[48] Wolin K, Steinberg D, Lane I, Askew S, Greaney M, Colditz G, Bennett G (2015). Engagement with eHealth Self-Monitoring in a Primary Care-Based Weight Management Intervention. PLoS One.

[49] Steinberg DM, Tate DF, Bennett GG, Ennett S, Samuel-Hodge C, Ward DS (2013). The efficacy of a daily self-weighing weight loss intervention using smart scales and e-mail. Obesity (Silver Spring).

[50] Spencer E, Appleby P, Davey G, Key T (2002). Validity of self-reported height and weight in 4808 EPIC-Oxford participants. Public Health Nutr.

